# The Impact of Vitamin D on Neuropsychiatric Disorders

**DOI:** 10.7759/cureus.47716

**Published:** 2023-10-26

**Authors:** Ghada A Wassif, Maram S Alrehely, Daliah M Alharbi, Asia A Aljohani

**Affiliations:** 1 Department of Anatomy and Embryology, Faculty of Medicine, Ain Shams University, Cairo, EGY; 2 Department of Anatomy, College of Medicine, Taibah University, Medina, SAU; 3 College of Medicine, Taibah University, Medina, SAU

**Keywords:** alzheimer's disease, parkinson's disease, schizophrenia, autism, depression, neuropsychiatric disorder, vitamin d deficiency

## Abstract

Vitamin D is a fat-soluble vitamin that has multiple biological effects on the body. Recent findings have also linked vitamin D deficiency to a range of neuropsychiatric disorders. The aim of this review article is to provide insight into the metabolism of vitamin D and its effect on the body, especially on the brain, and to recognize the role of vitamin D in some neuropsychiatric disorders. Vitamin D is well-known as a neuroactive steroid that modulates brain functions and development. There is strong evidence to show that optimal vitamin D levels are important to protect against neuropsychiatric disorders. Vitamin D has also been proposed to alter neurotransmitter pathways in the central nervous system. Abnormalities in these neurotransmitters have been implicated in various neuropsychiatric diseases, such as schizophrenia, Parkinson’s disease, and depression. Vitamin D also has some reported neurosteroid-like actions, including regulation of calcium homeostasis, clearance of amyloid-peptide, and antioxidant and anti-inflammatory effects, as well as possible protection against the neurodegenerative mechanisms associated with Alzheimer’s disease and autism. Vitamin D is an important modulator of brain development and has many functions in the brain. Several studies found that vitamin D has a protective role in neuropsychiatric disorders, and its supplementation decreases the development of these disorders and lowers their symptoms. Therefore, evidence shows that early intervention to maintain vitamin D concentrations at sufficiently high levels is crucial to slow, prevent, or improve neurocognitive decline.

## Introduction and background

It is widely acknowledged that vitamin D is a neuroactive steroid that affects a variety of brain functions. It is also crucial for brain growth, neurotransmission, neuroprotection, and immunomodulation, leading to alterations in brain neurochemistry and adult brain function [1,2].

Over the past 10-15 years, studies in which diet or vitamin D signaling has been manipulated in animal experiments have provided convincing evidence that this vitamin - more accurately referred to as a hormone - is required for normal brain homeostasis and development [3]. Vitamin D deficiency and insufficiency are global health issues that affect more than one billion children and adults worldwide [4]. The currently available literature on the Saudi Arabian population suggests that the rate of vitamin D deficiency is approximately 60% [5].

There is now a large body of epidemiological evidence showing that vitamin D deficiency is associated with a wide range of neuropsychiatric disorders and neurodegenerative diseases [6]. Early deficiencies have been linked to neuropsychiatric disorders, such as schizophrenia, and adult deficiencies have been associated with a host of adverse brain outcomes, including Parkinson’s disease (PD), Alzheimer’s disease (AD), depression, and cognitive decline [2].

For some disorders, such as epilepsy, multiple sclerosis, PD, and chronic stress, vitamin D may have some “neuroprotective” effects, according to a number of animal and clinical studies [7]. However, Harms et al. reported that vitamin D does have some influence on neurological or mental diseases, but it is probably a modest one, and vitamin D treatment is most likely to be used in conjunction with other therapies [7].

Early intervention in patients to improve vitamin D deficiency could also be important for health outcomes [6]. Furthermore, this simple and low-cost correction may contribute to the primo-secondary prevention of various neuropsychiatric disorders [8]. However, few individuals are aware of the effect of vitamin D in both early and late events in brain-cell growth and differentiation [3].

The aim of this review article is to provide an overview of the relationship between vitamin D deficiency and some neuropsychiatric disorders to increase awareness of this problem by describing the metabolism of vitamin D and its effect on the body. We review studies that have demonstrated the effects of vitamin D on brain development and adult brain function and recognize the role of vitamin D in some neuropsychiatric disorders, including AD, schizophrenia, depression, PD, and autism.

## Review

Vitamin D

Vitamin D is a fat-soluble vitamin that is derived from cholesterol. 1,25-Dihydroxy vitamin D (1,25(OH)2D) is the only biologically active form of vitamin D, which has two forms - vitamin D2 (ergocalciferol) and vitamin D3 (cholecalciferol) - and several metabolites [9,10].

The reference ranges of this important vitamin are described as deficiency at serum levels of <20 ng/mL, insufficiency at <30 ng/mL, sufficiency at <50 ng/mL, and toxicity at >150 ng/mL [10]. The global prevalence of vitamin D3 deficiency is 30%-90% [10].

Vitamin D synthesis and metabolism

The D vitamins are sterols with hormone-like functions. There are two main sources of preformed vitamin D activity: endogenous vitamin precursors such as 7-dehydrocholesterol, which is an intermediary in the production of cholesterol, and diet, such as cholecalciferol (vitamin D3) and ergocalciferol (vitamin D2), which are found in animal tissues and plants, respectively [11].

The D2 and D3 forms differ only in their side-chain structure. The differences do not affect metabolism by activation, and both forms function as prohormones [12]. The 7-dehydrocholesterol cutaneous precursor is converted into vitamin D3 (cholecalciferol) on the epidermis by ultraviolet B (UVB) rays with wavelengths of 295-310 nm, which includes two concurrent hydroxylations of active vitamin D3 [13].

The first hydroxylation occurs at 25 sites, which is catalyzed in the liver by the common 25-hydroxylase. The reaction product, 25-hydroxycholecalciferol (25-OH-D3) (calcidiol), is the vitamin D found in the blood and the main form of vitamin D storage [11]. The second hydroxylation is carried out by 1-hydroxylase, which is expressed in the kidney, prostate, lung, placenta, immune cells, and brain. 1-Hydroxylase produces 1,25-diOH-D3 (calcitriol) [13].

It is important to note that calcitonin, a thyroid hormone, controls kidney 1-hydroxylase. This thyroid hormone lowers blood calcium (Ca2+) by decreasing mobilization from bone, intestinal absorption, and renal reabsorption, working against the effects of parathyroid hormone. Active vitamin D plays a critical role in calcium/phosphorus homeostasis. The extra-renal enzyme is rigorously controlled by pro-inflammatory cytokines, and the active vitamin D generated regulates cell proliferation and differentiation in many tissues and organs, as well as in immune cells [11,13].

All enzymes contributing to the metabolism of vitamin D belong to the family of cytochrome P450 (CYP450), which is additionally engaged in oxidation/reduction reactions and drug catabolism in the liver [13]. The active molecule calcitriol binds to intracellular receptor proteins. The calcitriol-receptor complex interacts with response elements in the nuclear DNA of target cells and either selectively stimulates or represses gene transcription. The most prominent function of calcitriol is to control the levels of calcium and phosphorus in the serum [11].

In 2018, Anjum et al. [14] reported that vitamin D is becoming increasingly recognized as a necessary neuro-steroid with various actions in the brain. Circulating 25(OH) vitamin D crosses the blood-brain barrier and enters glial cells and neuronal cells to be converted into 1,25(OH)2D, which is the biologically active form of vitamin D [14]. Vitamin D is a powerful antiproliferative and prodifferentiating agent [15].

Structure and function of vitamin D receptor (VDR) in the body

Over the last two decades, research has demonstrated that particular changes in gene expression controlled by an intracellular VDR are responsible for the various biological actions of 1,25-dihydroxyvitamin D (1,25(OH)2D). When the VDR is activated directly by 1,25(OH)2D, it quickly binds to the target gene regulatory areas. From there, it starts to create massive protein complexes, the functional activities of which are crucial for direct transcription changes.

The VDR protein consists of three unique regions: a C-terminal ligand-binding action domain, an N-terminal dual zinc finger DNA-binding domain, and a large unstructured region connecting the two functional domains of the protein [16]. The function of ligand-activated VDR is to direct cellular transcription machinery to specific genomic sites where they can participate in RNA production by encoding proteins that are essential to certain biological processes. In this way, 1,25(OH)2D acts on intestinal and kidney epithelial cells, as well as some bone cells, to regulate the metabolism of minerals [16].

Vitamin D also controls gene networks involving the synthesis of bile acids in the colon, xenobiotic substance degradation in various tissues, keratinocyte differentiation in the skin, the formation and regeneration of dermal hair follicles, and the functions of the major cell types involved in both innate and adaptive immunity. The genes and gene networks defined as being responsible for these 1,25(OH)2D biological behaviors are extensive [16].

VDR in the brain

The VDR is widespread in brain tissue, and the biologically active form of vitamin D (calcitriol) has shown neuroprotective effects, including the clearance of amyloid plaques, thus playing an essential role in brain health [14,17]. The VDR exists in most neurons and some glia in adult rats and human brains. Its protein is present in the pontine-midbrain region, cerebellum, thalamus, hypothalamus, basal ganglia, hippocampus, and olfactory system, as well as the temporal, orbital, and cingulate cortices of both human and rat brains [7].

Therefore, the presence of metabolites of vitamin D, stimulating enzymes, and the VDR in the brain suggests that the vitamin D mechanism, similar to other neurosteroids, may play a role in sustaining normal brain function. In addition, high levels of VDR in the developing brain may suggest that vitamin D is involved in neurodevelopment [7].

Effects of vitamin D on brain development and adult brain functions

Cui et al. reported that accumulating evidence suggests that adequate vitamin D levels are needed for normal brain growth and the maintenance of healthy brain function [1].

Vitamin D and brain development

Studies conducted over the past 10 years have argued that vitamin D influences the developing brain via its effects on cell proliferation, neurotrophic factor production, cytokine control, neurotransmitter synthesis, intracellular calcium signaling, antioxidant activity, and the expression of genes/proteins that contribute to neuronal differentiation, structure, and metabolism. Dysfunction in any of these processes can lead to adverse development [18].

Vitamin D and adult brain functions

Several lines of evidence suggest that vitamin D plays a role in brain function and development. In 2011, Harms et al. [7] reported that vitamin D may decrease the effects of glucocorticoids, which are secreted by the adrenal glands in response to stress. It has also been shown that chronically high levels of glucocorticoids, in response to persistent stress, cause neuronal atrophy and subsequent cell death. In a hippocampus progenitor cell line, calcitriol showed complete inhibition of cell differentiation and partial inhibition of glucocorticoid receptor activity by fully antagonizing dexamethasone, which is a synthetic glucocorticoid [7].

In his review article, Holick [4] stated that vitamin D acts as a potent antioxidant through the inhibition of free-radical generation by nitric oxide synthase and gamma-glutamyl transpeptidase. He added that vitamin D appears to play an important role in vascular health in the brain, with anti-inflammatory activity [19].

Furthermore, vitamin D upregulates the production of several neurotrophic factors that promote the survival and function of neurons. It has been shown to modify a number of neurotrophic agents, such as nerve growth factor (NGF), which is important for the growth and survival of many cell types in the brain, particularly the cholinergic forebrain neurons that project to the hippocampus. Therefore, vitamin D modulates hippocampal development by increasing NGF production [17,20].

In addition, calcitriol can enhance the synthesis of glial cell line-derived neurotrophic factor (GDNF) mRNA in C6 glioma cells, which is integral to the development of the dopaminergic and noradrenergic systems. Vitamin D produces a neuroprotective effect by regulating NGF and GDNF. It also appears to provide some defense against exciting neurotransmitters, including glutamate [20].

Vitamin D deficiency and neuropsychiatric disorder

Alzheimer’s Disease

Definition: AD is a chronic, slowly progressing neurodegenerative disorder that begins well before its symptoms first appear. It is the most common cause of dementia seen in aging individuals, as it is responsible for 60%-80% of cases [21].

Epidemiology: Although there are no official statistics on the spread of AD in Saudi Arabia, experts estimate that there are at least 50,000 AD patients in the kingdom, most of whom are women [22].

Relationship between vitamin D deficiency and AD: There is growing evidence that hypovitaminosis D has played a role in the progression of AD [6]. Interestingly, studies have observed a substantially high prevalence of vitamin D insufficiency in AD patients [23].

Lu’o’ng et al. [23] stated that vitamin D deficiency is more common in elderly females with AD, which has been linked to low mood and impaired cognitive performance in older adults. There is also an association between mini-mental state examination (MMSE) test scores and serum 25OHD levels, where, in one study, patients with adequate levels of vitamin D had significantly higher MMSE scores compared to patients with inadequate levels of vitamin D [23]. The correlation between vitamin D-associated cellular pathways and neurodegeneration should be considered under three titles: oxidative stress prevention, neurotrophic factor regulation, and Ca2+ homeostasis.

Vitamin D, as an antioxidant, controls brain detoxification processes through the up-regulation of γ-glutamyl transpeptidase activity. It also regulates the synthesis of neurotrophic factors, which are essential for neuron fate and neuronal survival. In addition, calcium homeostasis affects many cellular mechanisms, particularly neurotransmission. Vitamin D was found to alter calcium uptake in some excitable cells by modulating L-type voltage-sensitive calcium channels in the hippocampus and by inducing the synthesis of calcium-binding proteins [24].

Additionally, calcitriol was suggested to alter cholinergic, dopaminergic, and noradrenergic neurotransmitter systems in the central nervous system (CNS) [25]. In some AD patients, vitamin D has been shown to clearly exhibit a beneficial role and improve cognitive function [23].

Positive effect of vitamin D supplementation: Gezen et al. [25] reported significant benefits in calcitriol treatment in AD-related cell culture and vitamin D supplementation in animal studies, including the prevention of amyloid-induced cytotoxicity via the induction of voltage-sensitive calcium channels and inducible nitric oxide synthase. Increased consumption of vitamin D and fish oil has been shown to prevent neurodegeneration [25].

Parkinson’s Disease

Definition: PD is the second most common form of neurodegeneration in the elderly population [26]. The disease is characterized by substantial degeneration of nigra dopaminergic neurons, leading to clinical symptoms, such as bradykinesia, resting tremor, and rigidity [27].

Epidemiology: PD affects one to two out of 1,000 people at any time. However, the prevalence of PD increases with age, as it affects 1% of those over the age of 60 [28]. The prevalence of PD in Saudi Arabia has been estimated to be 27 per 100,000 people [29].

Relationship between vitamin D deficiency and PD: Vitamin D affects the development, maintenance, and survival of neurons and plays a neuroprotective role in the CNS; its deficiency is associated with PD [30]. Lu’o’ng et al. [23] demonstrated that there is a significant association between vitamin D and PD; as low vitamin D levels may play an important role in the development or pathogenesis of PD, they suggest that elevated vitamin D levels might provide protection against PD [26]. Lv et al. reported that the distribution of VDRs is affected in the substantia nigra of PD patients, with the vitamin also playing a role in the regulation of tyrosine hydroxylase gene expression and, consequently, dopamine biosynthesis [27].

As PD motor symptoms increase, serum calcitriol concentration progressively decreases. Vitamin D was proposed to alter dopaminergic, cholinergic, and noradrenergic neurotransmitter pathways in the CNS. Furthermore, both neuronal plasticity and axogenesis may be influenced by vitamin D. Several studies have demonstrated that vitamin D protects the structure and integrity of neurons by improving the synthesis of neurotrophic factors and detoxification pathways [28].

Positive effect of vitamin D supplementation: A study done by Lu’o’ng et al. [23] showed that vitamin D may be beneficial in PD patients, as one patient showed improved rigidity and akinesia, and was able to decrease their levodopa dosage after vitamin D therapy [26].

Schizophrenia

Definition: Schizophrenia is recognized as a significant mental disorder with both positive symptoms (e.g., delusion and hallucination) and negative symptoms (e.g., blunted influence, and emotional and social withdrawal). These symptoms may adversely affect the psychological, occupational, or interpersonal functioning of an individual, and its consistency [31].

Epidemiology: Schizophrenia is a debilitating neurological illness that affects approximately 1% of the world’s population. The Ministry of Health of Saudi Arabia estimated that 22.4% of those treated at outpatient mental health facilities suffered from psychiatric and behavioral problems induced by schizophrenia and delusional problems [31].

Relationship between vitamin D deficiency and schizophrenia: Kinney et al. [32] mentioned that prenatal vitamin D deficiency is a significant etiological factor in schizophrenia. The incidence of vitamin D deficiency increases with latitude and cold weather because the skin’s exposure to UVB radiation from sunlight is the only natural source of vitamin D, where the decreased hours and strength of sunlight in higher latitudes make it impossible for people to obtain enough vitamin D, especially in winter months. Skin color also affects the absorption of UVB radiation from sunlight and one’s ability to synthesize vitamin D [32].

McGrath et al. added that there is a lack of adequate evidence to advise public health on the use of vitamin D for schizophrenia prevention. The literature currently lacks information on the critical window during which hypovitaminosis D affects brain function, and the processes that underlie the apparent nonlinear association between neonatal vitamin D and schizophrenia incidence are not well understood [15].

Chiang et al. conducted a case-control study of 69 individuals with first-episode psychosis and 69 healthy controls matched for age, gender, and ethnicity; they found severe vitamin D deficiencies in patients with first-episode psychosis [33].

Berridge reported that vitamin D plays an important role in regulating a variety of transmitters and their downstream signaling pathways, such as the development of dopaminergic neurons. Under vitamin D deficiency, changes are observed in the metabolism of dopamine (DA), which may reflect the characteristic decline in DA neuron expression present in schizophrenia [34].

In 2019, Lau et al. stated that there is little understanding of the genetic and environmental causes of schizophrenia, but many researchers are gradually finding connections between maternal vitamin D status and the risk of schizophrenia. They explained that vitamin D deficiency during development influences brain growth, and the VDR is found in areas of concern in schizophrenia (e.g., dopaminergic regions). Animal studies have shown that temporary prenatal vitamin D deficiency is consistent with neurochemical and behavioral changes [35].

Positive effect of vitamin D supplementation: McGrath et al. reported that prenatal vitamin D supplementation in women at risk of hypovitaminosis D reduced the incidence of schizophrenia in offspring [15].

Depression

Definition: Depression is a disorder characterized by depressive mood, or lack of motivation or enjoyment in almost all events, most of the day, for a duration of at least two weeks [36].

Epidemiology: Depression was estimated to be the most prevalent mood disorder at King Fahd Hospital University, Al Khobar, Saudi Arabia, with the majority of patients being female [37].

Relationship between vitamin D deficiency and depression: In 2015, Patrick et al. reported that depression may occur as a consequence of inflammation resulting from the inhibition of serotonin release, as serotonin plays an essential role in mood regulation. They added that serotonin also regulates a wide range of cognitive functions and social behaviors in addition to mood [38].

Berridge explained that depression is triggered by alteration in neuronal function arising from a rise in glutamate that activates stimulating neurons, which can be responsible for the decreased inhibitory function of gamma-aminobutyric acid-ergic neurons and their amount. This disparity between stimulating and inhibitory neurons will lead to the initiation of depression [39].

At the cellular level, the concentration of intracellular Ca2+ within inhibitory neurons is increased by an increase in entry through the N-methyl-D-aspartate receptors (NMDARs) and by activation of the phosphoinositide signaling pathway, producing inositol trisphosphate (InsP3), which releases Ca2+ from internal stores. The role of these two pathways in driving Ca2+ elevation is confirmed by the fact that stress can be alleviated by ketamine, which inhibits the NMDARs, and scopolamine, which inhibits the InsP3/Ca2+ pathway driving M1 receptors. Berridge also added that increased Ca2+ not only leads to depression but also explains why people with depression are at high risk of AD development [39].

In addition, Bersani et al. have demonstrated substantial correlations between low baseline levels of vitamin D and depressed symptoms over time, indicating that reduced vitamin D levels may play a role in the pathophysiology of these disorders. They added that vitamin D has been shown to be implicated in neurotransmission, neurogenesis, and the regulation of proinflammatory cytokines, as well as brain development [40].

Recently, Menon et al. reviewed several cross-sectional studies, a few cohort studies, and one case-control study and found that depressed people had lower vitamin D levels than those who were non-depressed (control groups), and those with the lowest vitamin D levels had the greatest risk of depression [41].

Positive effect of vitamin D supplementation: Berridge showed how vitamin D prevents the onset of depression by activating a number of processes, such as controlling the expression of mitochondrial proteins, antioxidant genes, and calcium homeostasis; controlling inflammation and the formation of serotonin; and controlling the epigenetic landscape by expressing demethylases, which are critical to maintaining normal healthy neurons [39]. Recently, Menon et al. added that many studies of randomized controlled trials suggest superior therapeutic benefits of vitamin D supplementation in clinical, rather than subsyndromal, depression [41].

Autism

Definition: Autism spectrum disorders (ASDs) are a heterogeneous group of complex biologically based neurodevelopmental disorders that usually appear in the first three years of life [42,43]. Multiple genetic and environmental risk factors contribute to the lifelong developmental disabilities known as autism, which are mostly incurable [42].

Individuals with autism suffer from poor verbal and nonverbal communication, as well as poor social interaction. The majority of neurobehavioral and cognitive disorders are connected to this illness. Its symptoms include a wide range of socio-communication problems, attention deficit hyperactivity, speech issues, seizure disorders, intelligence disabilities, fragile X syndrome, and tuberous sclerosis. In addition, some children may have a variety of mental health issues, such as anxiety or depression [42].

Epidemiology: Although the prevalence of autism has increased dramatically over the past two decades, the reason for this increase remains unclear. It is estimated that the prevalence of autism in Saudi Arabia is 18 out of 10,000 people, which is slightly higher than the 13 out of 10,000 people reported in developed countries [44]. The disorder is accompanied by mental retardation in three out of four patients, and boys are four times more likely than girls to have the disorder [45].

Relationship between vitamin D deficiency and autism: The underlying etiology of ASD is most likely multifactorial, and it is suggested that, in most cases, autism results from the interaction of multiple genetic and environmental factors. There are several known risk factors, including a variety of mutated and variant genes, advanced paternal age, exposure to toxins and medications in early development, prematurity, and birth complications [45].

Recently, maternal/neonatal vitamin D deficiency has been proposed as a possible environmental risk factor for ASD due to its involvement in early neurodevelopment, the immune system, and gene regulation processes [43]. Cannell reported that individuals with autism have immune function defects similar to those affected by vitamin D deficiency, such as elevated levels of the inflammatory cytokine. He added that much of the ongoing inflammation in autistic brains is the result of oxidative stress, where vitamin D has the most effective anti-inflammatory properties [46].

Regardless of the cause of the autoimmune inflammatory state, supplementing infants and children with vitamin D is very likely to help, as vitamin D upregulates the production of dendritic (peacemaker) lymphocytes, which decrease the intensity of autoimmune attack by upregulating interleukin-10, an anti-inflammatory cytokine [46]. Cannell added that the activated vitamin D hormone (calcitriol) protects brain tissue by reducing the levels of inflammatory cytokines, which, when elevated, are strongly associated with cognitive impairment in ASD [46].

Positive effect of vitamin D supplementation

Guo et al. revealed that serum levels of 25-(OH)2D were significantly lower in autistic children than in typically developing children. In addition, autistic children’s symptom scores were significantly reduced after vitamin D3 supplementation [47].

Does vitamin D improve neuropsychiatric disorders?

Vitamin D deficiency is very common in patients with neuropsychiatry, regardless of diagnostic type. As the role of vitamin D in psychiatric disorders remains unclear, further studies are required to better understand the pathophysiological function of vitamin D in neuropsychiatric disorders and to take advantage of vitamin D or synthetic VDR antagonists in neuropsychiatric care. As vitamin D is safe and cheap, it is, therefore, an attractive therapeutic to test its effectiveness in a wide range of mental health disorders [6].

Several studies have indicated that vitamin D supplementation should be measured while taking into account the baseline vitamin D status of each patient, as well as their particular ability to respond to vitamin D supplementation, as factors such as adiposity and VDR genotypes may affect this capability. Therefore, vitamin D supplementation may offer a potentially effective treatment avenue for neuropsychiatric patients, even if vitamin D shows only small effects on clinical outcomes [1].

## Conclusions

Vitamin D is an effective brain growth modulator and achieves many brain functions by influencing numerous regulatory processes. Several trials have recorded cognitive improvements in neuropsychiatric disorders with vitamin D supplementation. However, additional studies are required to fully understand the pathophysiological role of vitamin D in these disorders and take advantage of vitamin D or synthetic VDR antagonists as innovative therapeutic strategies in the treatment of neuropsychiatric disease. The considerations discussed above may encourage clinicians to correct hypovitaminosis D in patients.
